# Paramedian forehead flap in the treatment of nasal, non-melanoma skin cancer: a cross-sectional study

**DOI:** 10.1590/0100-6991e-20223034-en

**Published:** 2022-08-25

**Authors:** RENAN DIEGO AMÉRICO RIBEIRO, VITOR PENTEADO FIGUEIREDO PAGOTTO, GIULIA GODOY TAKAHASHI, RAFAEL MAMORU CARNEIRO TUTIHASHI, CRISTINA PIRES CAMARGO, FABIO DE FREITAS BUSNARDO, ROLF GEMPERLI

**Affiliations:** 1 - Hospital das Clínicas da Faculdade de Medicina da Universidade de São Paulo, Serviço de Cirurgia Plástica - São Paulo - SP - Brasil; 2 - Instituto do Câncer do Estado de São Paulo, Serviço de Cirurgia Plástica - São Paulo - SP - Brasil

**Keywords:** Skin Neoplasms, Nose Neoplasms, Nose Deformities, Acquired, Myocutaneous Flap, Quality of Life, Neoplasias Cutâneas, Neoplasias Nasais, Deformidades Adquiridas Nasais, Retalho Miocutâneo, Qualidade de Vida

## Abstract

**Introduction:**

nose is the central point of the face and vulnerable to the occurence of non-melanoma skin cancer (NMSC), impacting on appearance. The paramedian forehead flap (PMFF) is considered the best option to treat extensive nasal defects. The objective of this study is to present the experience on PMFF for nasal reconstruction in the treatment of NMSC of a cancer referral center.

**Methods:**

retrospective study was carried out through data from medical records of patients who underwent nasal reconstruction with PMFF due to NMSC at the Cancer Institute of the State of São Paulo (ICESP).

**Results:**

111 patients were identified, mostly ederly, with comorbidities and on initial tumors (T1 and T2). Basal cell carcinoma (BCC) was the predominant histological type. Dorsum and tip were the most affected subunitis. In addition to skin coverage, reconstruction of the lining and structural framework was also performed in half of the cases. Second intention healing was the technique of choice in closing the donor area. Pedicle division ocurred predominantly in the second operation and the median time to complete reconstruction was 6 months. There were low complication rates.

**Conclusions:**

the PMFF is safe and effective to treat nose NMSC, even in cases of high complexity. Since the treatment time can be prolonged and impact on quality of life, it is essential to emphasize and discuss this aspect with the patients before surgery.

## RESUMO

**Introdução:**

o nariz é o ponto central da face e vulnerável à ocorrência de câncer de pele não-melanoma (CPNM), com impacto potencial na aparência. O retalho frontal paramediano (RFPM) é considerado a melhor opção para o tratamento de defeitos extensos no nariz. O objetivo deste estudo é apresentar a experiência de um centro oncológico de referência no uso de RFPM para reconstrução nasal no tratamento do CPNM.

**Métodos:**

estudo retrospectivo foi desenvolvido através do levantamento de dados de prontuário de pacientes submetidos à reconstrução nasal com RFPM devido à CPNM no Instituto do Câncer do Estado de São Paulo (ICESP).

**Resultados:**

111 pacientes foram identificados, a maioria idosos, com comorbidades e portadores de tumores iniciais (T1 e T2). O carcinoma basocelular (CBC) foi o tipo histológico predominante. Dorso e ponta foram as subunidades mais acometidas. Além da cobertura cutânea, em metade dos casos foi realizada também a reconstrução do forro ou arcabouço do nariz. Cicatrização por segunda intenção foi a técnica de escolha no fechamento da área doadora. A liberação do pedículo foi realizada predominantemente no segundo tempo cirúrgico e o tempo para finalização do tratamento teve mediana de 6 meses. Houve baixa taxa de complicações.

**Conclusões:**

o RFPM é seguro e eficaz no tratamento do CPNM nasal, mesmo em casos de elevada complexidade. O tempo total de tratamento pode ser prolongado e impactar na qualidade de vida, sendo fundamental enfatizar e discutir este aspecto com o paciente antes da cirurgia.

## INTRODUCTION

The nose is the most central and prominent point on the human face. Its size, shape, and symmetry are fundamental in defining an attractive face^1^. Due to its prominent location and the delicacy of the skin that covers it, the nose is also particularly vulnerable to injuries of different natures. Trauma, infections, and neoplasms can disfigure it, compromising its function and appearance, with serious consequences for quality of life^2^.

Historically, the treatment of nasal deformities has been described and studied through victims of war wounds and infectious diseases^1^. In the contemporary world, however, non-melanoma skin cancer (NMSC) is highlighted as a cause of nose mutilation. NMSC was diagnosed in more than 1 million people worldwide in 2018^3^. In the United States, where this type of cancer is the most frequent, there are estimates of mortality of 4,500 people per year and an annual cost of US$ 8.1 billion to the health system^4^ ^-^ ^6^. In Brazil, 177,000 new cases and 2,000 deaths are estimated in 2020^7^.

The paramedian forehead flap (PMFF) is considered the best option for the treatment of extensive defects in the nose, providing similar skin and reliable vascularization^8^ ^,^ ^9^. However, factors such as age, comorbidities, and oncological status can negatively impact surgical outcomes. Due to the importance that NMSC is currently gaining as a cause of complex nasal defects that require reconstruction, it is important to know the clinical and surgical aspects that determine the success of treatment with this type of flap.

The aim of this study is to present the experience of a reference oncology center in the use of PMFF for nose reconstructions in the treatment of NMSC. This case series aims to reinforce the role of this technique in the management of complex nasal defects, demonstrating its safety and alerting to the pitfalls that can negatively impact results.

## METHODS

We conducted a retrospective study by collecting data from the medical records of patients who underwent nasal reconstruction with PMFF due to NMSC at the Cancer Institute of the State of São Paulo (ICESP) between January 1, 2011, and December 31, 2019. We excluded patients with incomplete data in medical records.

We collected data on epidemiological profile, comorbidities, histological type of skin cancer, staging, and follow-up time. We also gathered surgery-related information, such as affected subunits, nasal lining reconstruction or structural framework, interval until pedicle release, complications, total number of surgeries per patient, type of anesthesia, and time to completion of the reconstruction.

We obtained preoperative, intraoperative, and postoperative photographic records, as well as informed consent forms to perform the images. Photographic analysis allowed the identification of the number of nasal subunits affected by the neoplasm, as well as the other facial units involved by the same lesion, in addition to calculating the resected area. All photographic analyzes were performed using the Adobe^®^ Photoshop software.

We described data according to nature and distribution. Thus, we described nonparametric variables by percentage and interquartile range (IQR), and parametric ones, by mean and standard deviation. Nominal or dichotomous variables were presented by percentage of frequency. We performed the tests of Spearman Rank correlation coefficient between the variables affected subunits, lining or structural framework reconstruction, surgical time for pedicle release, complications, total number of surgeries per patient, and time to completion of the reconstruction.

The Ethics Committee for the Analysis of Research Projects of ICESP approved this study, following the Declaration of Helsinki and the Document of the Americas, under registration 1666/20.

## RESULTS

We identified 111 patients surgically treated for nasal NMSC and reconstructed with PMFF in the ICESP database between January 1, 2011, and December 31, 2019 (62 males and 49 females).

The median age of participants was 69 years (IQR 59 78). Most of the sample was represented by white-skinned individuals (100 patients - 90.1%). The median income was BRL 1,250.00 (IQR BRL 1,045.00 2,000.00). Regarding education, most patients had complete elementary school (64.9%), followed by complete high school (21.6%); 9.4% had not attended school.

The median of comorbidities presented by patients was three (IQR 2 4), the main ones being systemic arterial hypertension (64%), smoking (51%), diabetes (26%), and alcoholism (23%). The main histological type was basal cell carcinoma (BCC), representing 81.1% of cases, followed by squamous cell carcinoma (SCC) (14.4%) and basosquamous carcinoma (3.6%). As for staging, 36% (40/111) of cases were T1, 35.1% (39/111) were T2, 21.7% (24/111) were T3, and 6.3% (7/111), T4. There was also one case of tumor in situ (Tis). The median follow-up time was 2.92 years (IQR 1.1 5.21 years) (Table 1).

**Table 1:** Clinical-demographic characteristics.

Variable	Median or Value (%) / IQR 25-75%
Sex	
Male	62 (55.9%)
Female	49 (44.1%)
Age years)	69 / 59 - 78
Income (BRL)	1,250.00 / 1,045.00 2,000.00
Skin color	
White	100 (90.1%)
Brown	11 (9.9%)
Schooling	
Variable	Median or Value (%) / IQR 25-75%
Elementary School	72 (64.9%)
High School	24 (21.6%)
College	5 (4.1%)
None	10 (9.4%)
Comorbidities	3 / 2 4
Histological type	
BCC	90 (81.1%)
SCC	16 (14.4%)
Other	5 (4.5%)
Staging	
Tis	1 (0.9%)
T1	40 (36.0%)
T2	39 (35.1%)
T3	24 (21.7%)
T4	7 (6.3%)

IQR: interquartile range.

**Figure 1:**
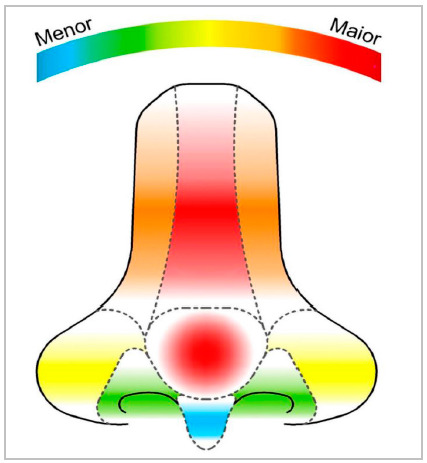
Graphic representation of nasal subunits by frequency of involvement. The main nasal subunits affected were dorsum (48.6%) and tip (46.8%), followed by lateral walls (43.2%) and wings (36%). Soft triangle (28.8%) and columella (15.3%) were less affected.

The average area affected by the neoplasm was 7.98cm². The median nasal subunits involved per patient was four (IQR 2.3 6.8). The main subunits involved were dorsum (48.6%) and tip (46.8%), followed by lateral walls (43.2%) and wings (36%). The soft triangle (28.8%) and the columella (15.3%) were less affected (Figure 1). In lesions that involved only lateral subunits, the use of PMFF ipsilateral to the defect was the preference in this series (67.3%).

**Figure 2:**
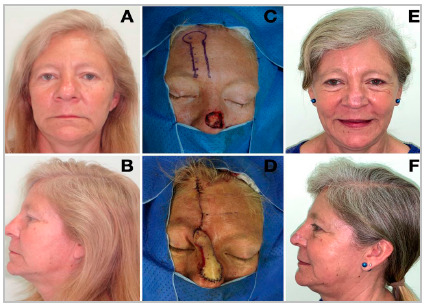
A 51-year-old woman with a history of smoking, without other comorbidities. She presented with a basal cell carcinoma involving mainly the nasal dorsum, but also part of the lateral walls and tip (A/B). The lesion was resected (C) and reconstructed with a paramedian forehead flap (D). Two flap weight reductions were performed. The pedicle was released after 66 days (E/F).

About half of the patients required lining reconstruction (49/111) (44.1%). The main techniques used were nasal septum chondromucosal flap (38.7%), nasolabial flap (28.6%), and folded frontal flap (12.2%). A free radial forearm flap was used in one case (2%). The structural framework was reconstructed in 34.2% of cases, mainly with conchal cartilage (52.6%) and septal cartilage (36.8%). Costal cartilage graft was performed in two patients (5.3%). Bone graft and PDS plate were also used in one patient each (2.6%). Most patients had the donor area treated for healing by second intention (75.7%). Complete primary synthesis of the donor area was performed in 26 patients (23.4%), while skin grafting was performed in only one (0.8%).

In addition to the nose, other facial units were affected by the same neoplasm in 28 patients (25.2%). Middle third (60.7%) and orbitopalpebral region (39.3%) were the most common. Lip involvement was identified in seven patients (25%) and one patient had facial nerve involvement. Almost half (42.8%) of the patients who had other facial units affected had these regions treated with a second pedicled flap. Another 39.2% were treated using the same forehead flap.

The pedicle was released in the second surgery in 80.2% of the patients and in the third surgery in 14.2%. The median time between performing the flap and releasing the pedicle was 52.5 days (IQR 35-98). About half of the patients (42.3%) completed the reconstruction with only two procedures, while 28.9% needed three. Another 24.3% needed additional surgical procedures. The median time to completion of the reconstruction was six months (IQR 3-24).

We identified complications related to the flap requiring re-approach in 14 (12.6%) patients. In the PMFF, we observed eight cases of partial necrosis (7.2%), one of total necrosis, two dehiscences, one hematoma, one scar retraction, and one nasocutaneous fistula. We identified complications in the donor area also in 14 (12.6%) patients, including skull exposure (5.4%), dehiscence (2.7%), necrosis (1.8%), infection (0.9%), unsightly scar (0.9%), and myiasis (0.9%). (Table 2)

**Table 2:** Surgical characteristics related to reconstruction.

Variable	Median or Value (Percentage) / IQR 25-75%
Lining reconstruction	49 (44.1%)
Chondromucous flap	19 (38.7%)
Nasolabial Flap	14 (28.6%)
Folded Forehead Flap	6 (12.2%)
Free Forearm Flap	1 (2.0%)
Other	9 (18.4%)
Structural reconstruction	38 (34.2%)
Conchal cartilage	20 (52.6%)
Septal cartilage	14 (36.8%)
Costal cartilage	2 (5.3%)
Other	2 (5.3%)
Variable	Median or Value (Percentage) / IQR 25-75%
Donor Area	
Second intention	84 (75.7%)
Primary closure	26 (23.4%)
Skin grafting	1 (0.8%)
Facial units affected beyond the nose	28 (25.2%)
Middle third	17 (60.7%)
Orbitopalpebral region	11 (39.3%)
Upper lip	8 (25.0%)
Pedicle release	52.5 days / 35 - 98 days
2^nd^ time	89 (80.2%)
3^rd^ time	16 (14.2%)
Others	6 (5.6%)
Completion of the reconstruction (months)	6 / 3-24
Flap complications	14 (12.6%)
Partial necrosis	8 (7.2%)
Total necrosis	1 (0.9%)
Dehiscence	2 (1.8%)
Hematoma	1 (0.9%)
Scar retraction	1 (0.9%)
Nasocutaneous fistula	1 (0.9%)
Donor area complications	14 (12.6%)
Skull exposure	6 (5.4%)
Dehiscence	3 (2.7%)
Necrosis	2 (1.8%)
Infection	1 (0.9%)
Unsightly scar	1 (0.9%)
Myiasis	1 (0.9%)
Reconstructed area	7.98cm²

## DISCUSSION

The projection of the nose in a plane anterior to the rest of the face makes this structure vulnerable to ultraviolet exposure, an important carcinogenic factor. Therefore, it is the place most affected by NMSC^10^. Similar to other large published series, individuals with nose NMSC in our series were predominantly men, elderly, and fair-skinned^11^ ^,^ ^12^. Cardiovascular diseases were the main comorbidities found, prevalent in a population with such characteristics. In agreement with the literature, the main histological type in this study was basal cell carcinoma (BCC), followed by squamous cell carcinoma (SCC)^11^.

Through a review of 420 patients, Sanniec et al. observed that the nasal subunits most involved by the neoplasm were the tip and wings^13^. In our sample, however, dorsum (48.6%) and tip (46.8%) were the most prevalent sites. The less affected subunits coincided, though, represented by the soft triangle (28.8% vs. 29%) and columella (15.3% vs. 10%). It is likely that these discrepancies are explained by differences in photoexposure, as the subunits located inferior to the nasal tip angle are relatively protected from the sun.

The main treatment modality for this neoplasm is surgical resection^14^ ^,^ ^15^, capable of causing extensive failure of nasal coverage, structure, and lining. The defect reconstruction may require tissue transfer, with the aim of repairing these three layers. The paramedian forehead flap is considered the best option for the treatment of these defects, especially when extensive, as it provides adequate coverage, reliable vascularization (based on the supratrochlear artery), and skin with characteristics similar to that of the nose^8^ ^,^ ^9^. Lesions in lateral subunits can be repaired using the pedicle ipsilateral or contralateral to the defect. In this study, there was a preference for the use of the ipsilateral base flap (67.3%). This option shortens the distance between the donor and recipient areas, allowing the creation of a smaller flap^16^ ^,^ ^17^. On the other hand, the contralateral base flap minimizes pedicle distortion and is related to lower long-term scar retraction^18^.

The local staging of NMSC is directly related to the size of the tumor^19^. Most of the population in this study was diagnosed with T1 and T2 tumors (36% and 35.1%, respectively), with a mean area of 7.98 cm² compromised by the neoplasm. Due to the exposed location, skin lesions in the nose are often identified by the patients themselves, with early diagnosis in the course of the disease^10^. When there is an extension of the neoplasm to other face parts, commonly the middle third, orbitopalpebral region, and lip, reconstruction becomes more challenging. For these cases, Menick proposed the use of a second or third flap, using different reconstructions for each unit^8^. In this study, the PMFF was used as a single reconstruction for all affected units in 39.2%. The breach of Menick’s principle is justified by the characteristics of this population, represented mostly by elderly individuals with multiple comorbidities, who benefit from less aggressive procedures.

T3 (20.7%) and T4 (6.3%) locally advanced tumors represented a considerable portion of this sample. In these cases, there is a greater chance of involvement of the nose deep structures^19^, which require joint reconstruction with skin coverage. If absent, the structural framework of the nose, formed by its osteofibrocartilaginous skeleton, must be redone. The objective is to shape and support the inner and outer layers of the nose, in addition to protecting the entire structure against scar retraction^12^. The repair of the nasal mucosa, which makes up the inner lining, must also be performed for adequate airway flow^13^. In this series, almost half of the patients required lining reconstruction (44.1%), generally with another flap. This number exceeds that of patients with T3 and T4 tumors. The explanation lies in the need for three-dimensional margins at the time of resection, which may include the deepest layers even in early tumors. On the other hand, scaffold reconstruction was necessary in 34.2%, using mostly autologous cartilaginous grafts from the ear and septum.

Traditionally, PMFF is performed in two steps. In the first, tissue transfer is carried out to close the defect, keeping the pedicle still connected to the donor area. After 20 days, when the vascularization of the flap becomes independent of the supratrochlear artery, its base is sectioned^20^ ^-^ ^23^. The longer interval between the creation of the flap and the release of the pedicle observed in our series can be attributed to several factors. Due to the complexity of the cases operated on in our service, there was a greater need for intermediate procedures before pedicle section, with an average of 3.12 surgeries per patient. There is also the logistical factor related to the great surgical demand of a public service of reference in oncological reconstructions.

Surgical retouches can be included when further refinements in nasal reconstruction are desired. To avoid flap necrosis, Burget and Menick recommend that additional procedures be performed before the pedicle section^24^. The technique in three or more steps has advantages over the classical technique. In addition to greater vascular safety due to the delay in disconnection of the supplying artery, it allows for serial weight loss of the flap in intermediate operations, providing a thin coverage at the end^24^ ^,^ ^25^. Furthermore, the three-step technique seems to provide a better aesthetic result at the end of the reconstruction^26^. More than half of the patients in this series had the reconstruction completed with at least three surgeries (57.7%), and a considerable proportion of patients needed at least four (24.3%).

The negative impact on the quality of life of patients undergoing multiple reoperations should be considered. Pagotto et al. demonstrated a statistically significant relationship between time to completion of reconstruction and quality of life of patients undergoing nasal reconstruction with PMFF. Patients who completed the reconstruction in a period of less than six months had better results in the quality-of-life domain of the FACE-Q questionnaire compared with the others (p=0.002)^27^. In our sample, the median time to completion of the reconstruction was six months (IQR 3-24). This long period is related to the complexity of the cases and the aforementioned logistical factors. Nonetheless, most patients had the pedicle released in the second procedure (80.2%), even when performed in three or more stages. Although contrary to what was established by Burget and Menick, the adoption of this flow shortened the patient’s discomfort and provided better quality of life, without increasing the rate of flap loss.

The preparation of the frontal flap was performed under general anesthesia in almost all patients. The author’s preference for general anesthesia is also defended by Menick, since the infiltration of the donor and recipient areas distorts the contour and makes an accurate assessment of the result impossible16. Only one case was performed with local anesthesia and sedation, due to the high surgical risk. Despite this, a previous study demonstrates that PMFF can be safely performed under general anesthesia in elderly individuals, with no statistically significant difference in the rate of postoperative complications when compared with younger patients. Subsequent procedures were preferably performed under local anesthesia, like proposed by Sanniec et al.^13^. Pedicle release and other minor refinements require less precision and are well tolerated by the patient under local anesthesia.

Several previous studies have shown a low rate of complications in PMFF. In the largest series published to date, Rohrich et al. recorded only 16 cases of complications requiring reoperation in a population of 1,334 patients^11^. Necrosis was the main cause in all series^11^ ^,^ ^13^. The incidence of complications in this series was similarly low, partial necrosis being the most common. There was only one case of total necrosis, which was treated with debridement and a new PMFF from the opposite side. Donor area complications occurred in 14 patients, although only two cases required reoperation. Skull exposure and dehiscence were the most common. Considering the total number of surgeries performed in our sample (347), only 16 were performed to treat complications.

The limitations of this study include its cross-sectional format and the sample selected. The inclusion of a population treated in a cancer center of reference in the public health system can select more complex cases. Due to the retrospective design of the study, we did not perform sample size calculation. The inclusion of only 111 patients may also impact the results of this study, although this is the largest series of its kind carried out in Brazil.

## CONCLUSION

Nasal reconstruction with a paramedian forehead flap is safe and effective in the treatment of non-melanoma skin cancer, even in highly complex cases. Factors related to the area to be reconstructed and the patient must be considered, including location, number of affected subunits, age, and comorbidities. Multiple steps may be necessary for the proper reconstruction. The total treatment time can be prolonged and impact quality of life, and it is essential to emphasize and discuss this aspect with the patient before surgery.
